# Identification of Germline *FOXE1* and Somatic *MAPK* Pathway Gene Alterations in Patients with Malignant Struma Ovarii, Cleft Palate and Thyroid Cancer

**DOI:** 10.3390/ijms25041966

**Published:** 2024-02-06

**Authors:** Carolina Pires, Ana Saramago, Margarida M. Moura, Jing Li, Sara Donato, Inês J. Marques, Hélio Belo, Ana C. Machado, Rafael Cabrera, Thomas G. P. Grünewald, Valeriano Leite, Branca M. Cavaco

**Affiliations:** 1 Unidade de Investigação em Patobiologia Molecular (UIPM), Instituto Português de Oncologia de Lisboa Francisco Gentil (IPOLFG), 1099-023 Lisboa, Portugal; cs.pires@campus.fct.unl.pt (C.P.); aasaramago@ipolisboa.min-saude.pt (A.S.); mmoura@ipolisboa.min-saude.pt (M.M.M.); ines.marques@nms.unl.pt (I.J.M.); bc-hbelo@ipolisboa.min-saude.pt (H.B.); vleite@ipolisboa.min-saude.pt (V.L.); 2 NOVA Medical School (NMS)-Faculdade de Ciências Médicas, Universidade Nova de Lisboa, 1169-056 Lisboa, Portugal; 3 Hopp Children’s Cancer Center (KiTZ), 69120 Heidelberg, Germany; j.li@kitz-heidelberg.de (J.L.); t.gruenewald@kitz-heidelberg.de (T.G.P.G.); 4 Division of Translational Pediatric Sarcoma Research, German Cancer Research Center (DKFZ), German Cancer Consortium (DKTK), 69120 Heidelberg, Germany; 5 National Center for Tumor Diseases (NCT), NCT Heidelberg, a Partnership between DKFZ and Heidelberg University Hospital, 69120 Heidelberg, Germany; 6 Serviço de Endocrinologia, Instituto Português de Oncologia de Lisboa Francisco Gentil (IPOLFG), 1099-023 Lisboa, Portugal; saramldonato@gmail.com; 7 Serviço de Anatomia Patológica, Instituto Português de Oncologia de Lisboa Francisco Gentil (IPOLFG), 1099-023 Lisboa, Portugal; aicunha@ipolisboa.min-saude.pt (A.C.M.); racabrera@ipolisboa.min-saude.pt (R.C.); 8 Institute of Pathology, Heidelberg University Hospital, 69120 Heidelberg, Germany

**Keywords:** thyroid cancer, malignant struma ovarii, cleft palate, thyroid ectopy, genomics, molecular tumour pathology, *FOXE1*, next-generation sequencing, *BRAF*, *AXIN1*

## Abstract

Germline variants in the FOXE1 transcription factor have been associated with thyroid ectopy, cleft palate (CP) and thyroid cancer (TC). Here, we aimed to clarify the role of *FOXE1* in Portuguese families (F1 and F2) with members diagnosed with malignant struma ovarii (MSO), an ovarian teratoma with ectopic malignant thyroid tissue, papillary TC (PTC) and CP. Two rare germline heterozygous variants in the *FOXE1* promoter were identified: F1) c.-522G>C, in the proband (MSO) and her mother (asymptomatic); F2) c.9C>T, in the proband (PTC), her sister and her mother (CP). Functional studies using rat normal thyroid (PCCL3) and human PTC (TPC-1) cells revealed that c.9C>T decreased *FOXE1* promoter transcriptional activity in both cell models, while c.-522G>C led to opposing activities in the two models, when compared to the wild type. Immunohistochemistry and RT-qPCR analyses of patients’ thyroid tumours revealed lower FOXE1 expression compared to adjacent normal and hyperplastic thyroid tissues. The patient with MSO also harboured a novel germline *AXIN1* variant, presenting a loss of heterozygosity in its benign and malignant teratoma tissues and observable β-catenin cytoplasmic accumulation. The sequencing of the F1 (MSO) and F2 (PTC) probands’ tumours unveiled somatic *BRAF* and *HRAS* variants, respectively. Germline *FOXE1* and *AXIN1* variants might have a role in thyroid ectopy and cleft palate, which, together with MAPK pathway activation, may contribute to tumours’ malignant transformation.

## 1. Introduction

Thyroid cancer (TC) is the most commonly occurring endocrine malignancy, accounting annually for ~3% of all cancer diagnoses [1]. Papillary thyroid cancer (PTC), a carcinoma of follicular cell origin, accounts for 80–85% of thyroid malignancies [2].

Struma ovarii (SO) is a rare ovarian teratoma characterised by the presence of ectopic thyroid tissue in more than 50% of the tumour. Malignant transformation is rare (about 0.1–5% of the cases), and the most common associated malignancy is PTC [3]. Due to its rarity, the genetic basis of malignant struma ovarii (MSO) remains poorly understood [4]. 

FOXE1 is a thyroid-specific transcription factor that, in conjunction with PAX8, HHEX and NKX2-1, maintains the differentiated state of the thyroid gland. Together with the other three transcription factors, FOXE1 is also a key player in thyroid organogenesis, as its expression during early thyroid development is required for thyrocyte precursor migration [5,6,7,8]. In the differentiated thyroid, FOXE1 is a transcriptional activator of the thyroperoxidase and thyroglobulin genes and mediates the ability of cells to respond to external stimuli [9]. 

Genetic studies have identified germline allelic variation in and near *FOXE1* to be strongly associated with the thyroid cancer risk, including single-nucleotide variants rs965513[A] (56 kb upstream of *FOXE1*) [10,11,12] and rs1867277[A] (within its promoter) [13], as well as variations within the *FOXE1* polyalanine tract [14], which has a variable length ranging from 11 to 22 alanine residues. Tomaz et al. also found compelling evidence of an association between some *FOXE1* variants and the familial thyroid cancer risk [15]. In addition, our group identified a rare *FOXE1* germline variant (p.Ala248Gly) in one family with three cases affected by TC [16]. Moreover, homozygous loss- and gain-of-function *FOXE1* variants [17,18,19,20,21,22] have both been implicated in Bamforth–Lazarus syndrome, characterised by congenital hypothyroidism, due to thyroid hypoplasia, ectopia or agenesis, cleft palate (CP), spiky hair with or without choanal atresia and bifid epiglottis [18,23]. 

Altered FOXE1 expression has been associated with PTC clinicopathological parameters, such as extra capsular invasion, lymph node metastasis and staging [24]; however, there is still no consensus on this matter [25]. Moreover, FOXE1’s expression levels have been positively associated with the degree of thyroid cancer differentiation, as less differentiated tumours such as anaplastic thyroid cancers present low or null FOXE1 expression [26]. Overall, the landscape emerging from the literature strongly indicates that either due to genetic variants or to other still unidentified regulators, the expression level of FOXE1 is a possible determinant underlying the susceptibility to PTC and/or other cancer phenotypes [27]. 

In this work, we describe and characterise two very rare germline *FOXE1* promoter variants, identified in two families with members having CP, MSO and TC, and report additional germline and somatic variants that may also be involved in the development of patients’ thyroid lesions.

## 2. Results

### 2.1. Identification of FOXE1 Promoter Variants in Two Portuguese Families

**Figure 1:**
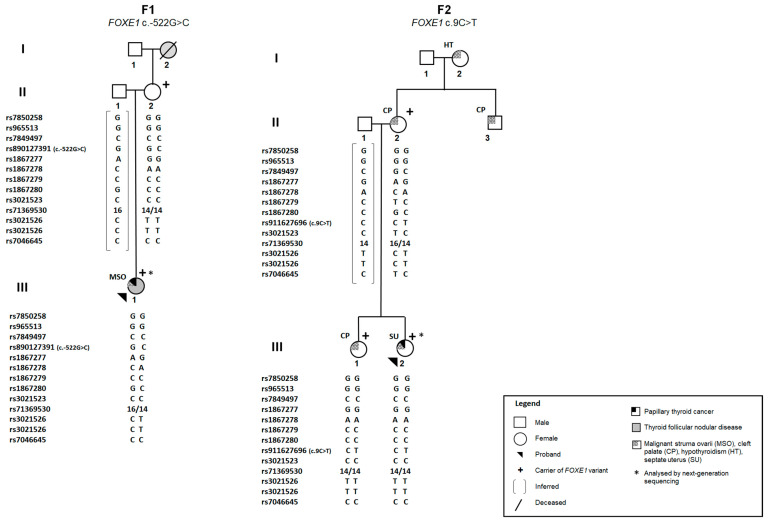
Pedigree of the two families analysed in this study. Family 1 (F1) harbours the *FOXE1* c.-522G>C variant and family 2 (F2) the *FOXE1* c.9C>T variant. Segregation analysis of single-nucleotide polymorphisms (SNPs) and rare variants from the *FOXE1* locus in the family members are shown.

The *FOXE1* gene is primarily expressed in the thyroid gland, being weakly detected in the adult testis and other tissues, such as the heart and tonsil [https://www.proteinatlas.org/ENSG00000178919-FOXE1/tissue (accessed on 24 January 2024)] [5]. The role of *FOXE1* in thyroid precursor cells’ migration, during embryogenesis, and its involvement in thyroid dysgenesis, thyroid ectopy, cleft palate and thyroid tumour development, prompted us to analyse this gene in two Portuguese families (F1 and F2) with members diagnosed with MSO (F1), TC (F2) and CP (F2). Two distinct germline *FOXE1* promoter variants were identified in the probands from families F1 and F2 (Figure 1). 

Patient III.1, from F1, diagnosed with MSO (PTC) and thyroid follicular nodular disease (FND), harboured the *FOXE1* c.-522G>C germline variant, in heterozygosity. This variant, rs890127391, has already been described in population databases, being very rare [minor allele frequency in the Non-Finnish European population (MAF_gnomAD_NFE_) < 0.01%], and was also identified in the proband’s mother (II.2), who was asymptomatic. It was not possible to investigate the presence of the variant in the proband’s grandmother (I.2), who presented FND, since she was already deceased. In family F2, another rare (MAF_gnomAD_NFE_ < 0.01%) *FOXE1* variant, c.9C>T (rs911627696; p.Ala3=), was identified in heterozygosity in the proband (III.2), who had PTC and a septate uterus, and was also detected in the sister (III.1) and mother (II.2), both with CP. There was no DNA available to study the maternal uncle (II.3) with CP and maternal grandmother (I.2) with hypothyroidism. Both *FOXE1* variants were absent in 100 Portuguese healthy controls and were not described on ClinVar nor in the literature. Additional single-nucleotide polymorphisms (SNPs), located centromeric to *FOXE1*, at the *FOXE1* promoter and at the 3′UTR region, were also genotyped (Figure 1). These included SNPs in the *FOXE1* locus, which have been reported to be significantly associated with the thyroid cancer risk: rs965513 (G>A), rs1867277 (G>A) and the polyalanine repeat region (rs71369530) [13,14,15], as well as rs7850258 (A>G), for which the G allele has been associated with an increased risk for cleft lip, cleft palate and hypothyroidism, while the A allele was associated with thyroid cancer [28]. In families F1 and F2, the analysed affected family members were homozygous for the G alleles of rs7850258 and rs965513 and did not carry the thyroid-cancer-associated A alleles (MAF_gnomAD_NFE_ = 34% for both SNPs). The rs1867277 thyroid cancer risk allele (A; MAF_gnomAD_NFE_ = 40%) was either absent or detected in heterozygosity in the F2 and F1 probands, respectively. Regarding *FOXE1* polyalanine tract expansions, *FOXE1^16Ala^* has been associated with PTC [14,15]. In the present study, the 16-Ala allele (MAF_gnomAD_NFE_ = 20–40%) [14,15,29,30] was detected in heterozygosity in the patient with MSO (F1, III.1), as well as in one family member with CP (F2, II.2) (Figure 1). 

### 2.2. Analysis of the Effect of FOXE1 Variants on FOXE1 Promoter Activity In Vitro

Dual-luciferase reporter assays were undertaken to investigate whether the identified *FOXE1* variants had an impact on its promoter transcriptional activity. Two different cell models, rat normal thyroid cells (PCCL3) and human PTC cells (TPC-1), were transfected with constructs carrying the wild-type (WT) or mutated *FOXE1* promoter sequence, upstream of the gene encoding firefly luciferase. 

**Figure 2:**
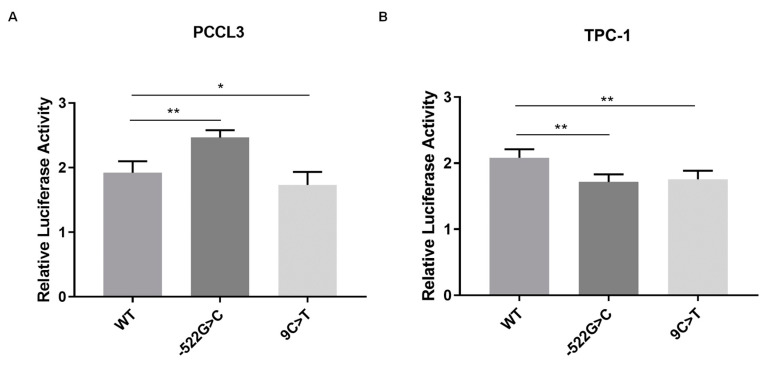
Effect of wild-type (WT) versus mutant *FOXE1* (c.-522G>C or c.9C>T) on *FOXE1* promoter activity. Promoter constructs were transfected in PCCL3 (**A**) and TPC-1 (**B**) cells. Values are expressed as fold of the empty vector. Data represent the mean ± standard deviation of at least three independent experiments, each in triplicate. *, *p* ≤ 0.05; **, *p* ≤ 0.01.

Briefly, in the PCCL3 normal thyroid cell model, the c.-522G>C variant, detected in the patient with malignant struma ovarii, led to an increase in promoter activity, when compared to the WT (*p* = 0.0064), while the c.9C<T variant, identified in the PTC/cleft palate family, led to a decrease (*p* = 0.0398). In the TPC-1 tumour cell model, both variants led to a decrease in *FOXE1* promoter activity, compared with the WT (*p* < 0.005) (Figure 2A,B). Thus, c.9C>T seems to be a loss-of-function variant, leading to lower FOXE1 promoter transcriptional activity in both normal and tumour cells. On the other hand, the c.-522G>T variant appears to have different impacts on *FOXE1* promoter activity depending on the thyroid cell (normal versus tumour) context.

### 2.3. Analysis of FOXE1 mRNA Expression in Patients’ Thyroid Tissue by RT-qPCR

Then, we evaluated whether *FOXE1* promoter variants led to differences in gene expression in vivo. *FOXE1* mRNA expression was assessed in the tumours and matched adjacent normal tissue from the F1 and F2 probands and three unrelated thyroid cancer cases from our cohort. This analysis was also performed in hyperplastic tissue from the patients presenting thyroid FND. 

**Figure 3:**
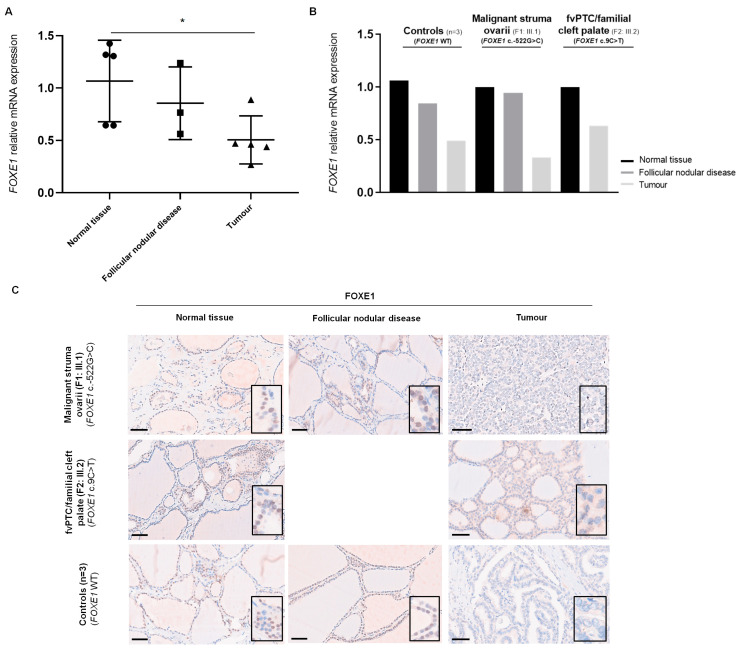
FOXE1 mRNA and protein expression in patients’ thyroid tissue. (**A**) mRNA expression levels of *FOXE1* in tumour and adjacent non-tumour (FND and normal) tissue were examined by RT-qPCR. Data represent the mean of triplicate experiments normalised to *YWHAZ* gene expression. (**B**) represents the results of *FOXE1* mRNA expression levels according to the case/group (controls’ tumour samples; malignant struma ovarii; and fvPTC/familial cleft palate). (**C**) Immunohistochemical staining of FOXE1 in patients’ tumours, hyperplastic (FND) and normal tissue sections. All images include a high-power view, showing positive (brown) or negative (blue) expression. Scale bar 60 µm. FND, follicular nodular disease; RT-qPCR, quantitative reverse transcription PCR; *, *p* ≤ 0.05.

Overall, the average mRNA expression levels of *FOXE1* were significantly lower in tumour tissue compared with adjacent benign thyroid tissue, regardless of the *FOXE1* mutation status. The hyperplastic tissues from the patients with FND revealed an intermediate degree of *FOXE1* expression, between those of normal and tumour tissues (Figure 3A), consistent with a decrease in the *FOXE1* expression levels throughout the tumourigenic process. The individual analysis of the control tumours, the MSO (family F1) and the fvPTC/familial cleft palate (family F2) in relation to the corresponding normal tissue (Figure 3B) supported the observations mentioned above. 

### 2.4. Analysis of FOXE1 Protein Expression in Patients’ Thyroid Tissue by Immunohistochemistry

Immunohistochemistry (IHC) was performed to analyse FOXE1 protein expression in FFPE tissues from the same patients studied by RT-qPCR. FOXE1 staining was negative in all tumour samples, regardless of the FOXE1 mutation status of the patient. A higher degree of FOXE1 expression was observed in matched adjacent normal thyroid and FND tissues, which showed positive and focal FOXE1 staining, present in cell nuclei (Figure 3C; Table S1). Thus, FOXE1 expression seemed to be downregulated both at the RNA and protein levels in tumours, independently of the presence of variants in the gene promoter, which parallels the progressive dedifferentiation in thyroid cancer development.

### 2.5. Next-Generation Sequencing Analyses of the Probands from Families F1 and F2

In addition to the identified *FOXE1* germline variants, we performed next-generation sequencing (NGS) of the F1 and F2 probands’ leukocyte and tumour nucleic acids to investigate whether additional molecular alterations could be involved in the initiation/progression of these patients’ tumours. 

Overall, no genetic fusions were identified in the tumour samples. A previously unreported missense germline *AXIN1* variant (c.1121C>G, p.Thr374Arg) was detected in the F1 proband, which, similarly to the *FOXE1* promoter variants, is classified as a variant of uncertain significance (VUS), according to the American College of Medical Genetics and Genomics (ACMG) guidelines (Table 1 and Table S2). This variant was also present in the asymptomatic proband’s mother. Variants in *AXIN1* are found in undifferentiated thyroid tumours [31,32]. This gene is an inhibitor of the Wnt signalling pathway, being responsible for the formation of the β-catenin destruction complex, preventing it from entering the nucleus [33]. Disruption of this complex can lead to the excessive accumulation of β-catenin. Increased cytoplasmic and, principally, nuclear β-catenin expression is usually suggestive of Wnt pathway dysregulation [34]. Since the F1 proband harboured a germline *AXIN1* variant, we decided to evaluate β-catenin expression by immunohistochemistry, in the same patients’ tissues that were analysed for FOXE1 protein expression (Table S3). Our results showed that none of the five PTCs presented nuclear β-catenin expression (0%). Instead, all the tumours, similarly to the normal and hyperplastic thyroid tissue, had membranous β-catenin expression (100%). The malignant struma ovarii and adjacent benign teratoma also presented cytoplasmic β-catenin expression, and, interestingly, loss of heterozygosity (LOH), a common genetic event in cancer development, known to be involved in the somatic inactivation of wild-type alleles from tumour suppressor genes, was observed, in the *AXIN1* gene, in the ectopic tissues from this patient (Figure S1A). It is possible that the LOH was associated with the observed β-catenin accumulation in the benign and malignant ovary teratoma, which was not seen in the eutopic thyroid tissue from the same patient (Figure S1A). Nevertheless, in this case, the *FOXE1* germline variant and co-occurring *AXIN1* inactivation did not appear to be sufficient for tumour initiation. Notably, this patient presented a rare somatic missense *BRAF* variant (c.1406G>C, p.Gly469Ala) that was only present in the malignant struma ovarii tumour, being absent in the adjacent (benign) teratoma tissue and in its normal and hyperplastic eutopic thyroid tissues (Figure S1B; Table 1 and Table S4). Therefore, oncogene activation seemed to be necessary to drive the tumour’s malignant transformation in the MSO patient. This variant is classified as deleterious in 10/14 in silico prediction software programs (Table 1), having already been reported in other malignant struma ovarii cases [35,36,37], but is rarely found in carcinomas of the thyroid gland. This patient also harboured a variant in the *TERT* promoter (*TERTp*), which, although classified as a polymorphism (MAF_gnomAD_NFE_ of ~30%), is associated with an increased risk of cancer [38] (Table 1 and Table S2). Additionally, we detected a gain at chromosome 3, where the *RAF1*, *CTNNB1*, *PIK3CA* and *DCUN1D1* genes are located (Table 1).

In the F2 proband with fvPTC and a septate uterus, a rare somatic *HRAS* variant (c.182A>G, p.Gln61Arg) was identified. This variant, already reported in thyroid cancer [39], is described as deleterious by most in silico prediction tools (Table 1 and Table S4). 

**Figure 4:**
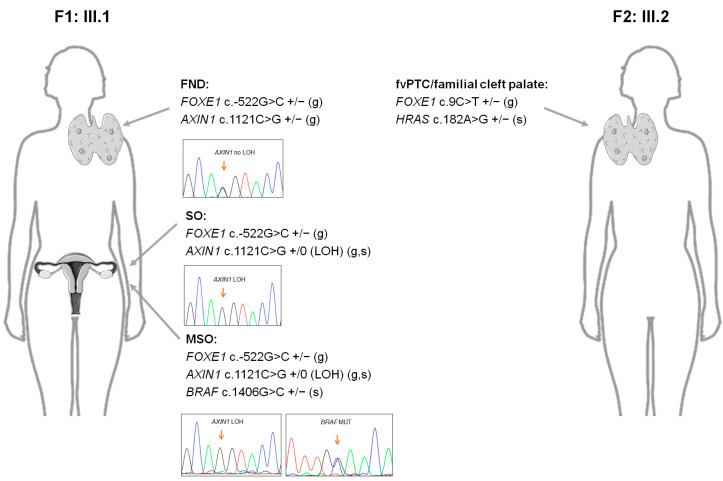
Schematic representation of the main genetic alterations identified in the probands from family 1 (F1) and family 2 (F2). Parts of the figure were obtained from Servier Medical Art (https://smart.servier.com/). FND, thyroid follicular nodular disease; SO, benign struma ovarii; MSO, malignant struma ovarii; fvPTC, follicular variant of papillary thyroid carcinoma; LOH, loss of heterozygosity; MUT, mutation; +/0, hemizygous variant; +/−, heterozygous variant; g, germline; s, somatic.

Our main findings in the probands from families F1 and F2 are summarised in Table 1 and Figure 4. 

## 3. Discussion

Malignant struma ovarii (MSO) is a very rare disease, with only around 200 cases reported in the literature. Thus, MSO’s carcinogenic mechanism is still mostly unknown, and no standard treatment has been established [4]. Currently, molecular data are available for approximately 50 MSO cases, and the associated somatic alterations are similar to those found in carcinomas of the thyroid gland [37]. Nevertheless, there has been little progress in the discovery of inherited genetic factors modulating the risk for MSO, as only one germline *KIT* mutation has been detected by NGS in a PTC arising in struma ovarii [40]. There are different hypotheses for the origin of teratomas, with some stating that totipotent primordial germ cells may miss their target destination [41], while, for others, struma ovarii represents ectopic thyroid tissue not through embryologic fragmentation or abnormal migration but from direct thyroid tissue differentiation from primary ovarian embryologic sources [42]. 

FOXE1 is a transcription factor, belonging to the FOX family, and plays important roles in thyroid precursor cells’ migration during embryogenesis and in normal thyroid development [5]. In animal models, Foxe1 is the only gene known to be associated with thyroid ectopy [6]. In addition, multiple studies have also suggested that rare variants and SNPs in the *FOXE1* gene locus are strongly associated with thyroid cancer (both familial and sporadic) and cleft palate [10,13,15,16,28,43]. 

In this study, we describe two families with cases of thyroid ectopy (malignant struma ovarii), thyroid cancer and cleft palate. In both families, two very rare, previously unpublished, germline *FOXE1* promoter variants were identified: F1) c.-522G>C, in the patient with MSO and FND and her asymptomatic mother; and F2) c.9C>T, in the proband with fvPTC and a septate uterus and her sister and mother with CP. 

Functional studies to evaluate the impact of these variants in *FOXE1* promoter activity showed that they had opposite effects in normal thyroid cells, with the variant identified in F1 leading to an increase in promoter activity, and the one identified in F2 leading to a decrease. These results suggest that these germline promotor variants, accounting for the deregulation of FOXE1 expression (both under- and overexpression), could have a role in thyroid ectopy and cleft palate. In the PTC cell model, both variants decreased *FOXE1* promoter transcriptional activity. Inherent neoplastic and genetic alterations (*RET/PTC1*) in these cells might have contributed to this similar effect. Thus, c.9C>T appears to be a loss-of-function variant, similarly to others that have been described in the *FOXE1* coding region, in patients with partial or complete Bamforth–Lazarus syndrome [17,18,19,20,21,22]. As regards the development of the Müllerian duct and, in particular, the septate uterus aetiology, further evidence is needed to establish a connection with the present genetic findings. On the other hand, c.-522G>C seems to be modulated by the thyroid cellular context. 

The recruitment of distinct transcriptional complexes, depending on the *FOXE1* variant(s) allele(s), has been proposed as a mechanism to explain *FOXE1*’s association with the thyroid cancer risk, with rs1867277 A allele leading to the enhanced activation of the *FOXE1* promoter [13], while rs7850258 A allele showing lower enhancer activity than the G allele, in vitro [28]. In addition, several studies have focused on assessing whether a correlation exists between SNPs/haplotypes and FOXE1 expression in thyroid carcinomas [44], but the results are not always in agreement. While Bychkov et al. found that nuclear FOXE1 expression in tumour cells, in the vicinity of the PTC border, was associated with the presence of the rs1867277 risk allele [45], other authors have found no significant correlation between the rs1867277 and rs965513 genotypes and the expression levels of FOXE1 [44,46]. Our results revealed lower *FOXE1* mRNA levels and absent protein expression in the patients’ thyroid tumours, when compared to matched adjacent normal and hyperplastic thyroid tissues, regardless of the *FOXE1* mutation status, which is in agreement with data from The Cancer Genome Atlas (TCGA) [47,48]. The loss of thyroid-specific proteins and differentiation markers is common in thyroid carcinogenesis. Specifically, absent FOXE1 expression is observed in most anaplastic thyroid cancers (ATCs) [26,49,50]. In addition to genetic factors, epigenetics may also explain the lower FOXE1 expression in PTC tissues, as the hypermethylation of the *FOXE1* promoter has been observed in other cancers [28], showing an inverse correlation with its expression [51]. 

Although the c.-522G>C germline *FOXE1* variant led to an increase in target gene expression in normal thyroid cells in vitro, the presence of this variant did not seem to be enough to drive thyroid tumourigenesis in this patient, who only presented FND, or in her mother, who was asymptomatic. Accordingly, Foxe1 overexpression in mice causes the development of FND but does not promote carcinogenesis [52]. 

In addition to underlying germline driver alterations, the somatic activation of MAPK-pathway-related oncogenes is likely required in thyroid cancer’s multistep development, as shown in familial thyroid cancer cases [53,54,55,56,57], particularly in a previous study from our group, in which we reported the identification of the *BRAF* p.Val600Glu in thyroid tumours from a family harbouring the rare germline *FOXE1* p.Ala248Gly variant [16]. Here, we detected a likely pathogenic *HRAS* variant in the PTC (encapsulated follicular variant) from patient III.2 (family F2), thus representing an *RAS*-like malignancy. Patient III.1 (family F1) with MSO revealed a more complex genetic background. Together with *FOXE1* c.-522G>C, a previously unreported heterozygous germline variant in the *AXIN1* gene was also identified. The *AXIN1* tumour suppressor is an inhibitor of the Wnt signalling pathway, which plays critical roles in embryonic development and adult tissue homeostasis. *AXIN1*, together with other genes, such as *APC*, forms a “destruction complex”, responsible for degrading β-catenin [33]. It is well established that there is a correlation between the subcellular localisation of β-catenin and cancer progression [58]. In hyperfunctioning adenomas and normal thyroid tissues, β-catenin is localised in the plasma membrane. In PTC, some cases present membranous expression, while others show the accumulation of β-catenin in the cytoplasm. In a study of 167 PTCs, only membranous β-catenin expression was detected [59]. Nuclear accumulation seems to be specific to familial adenomatous polyposis-related cribriform–morular thyroid carcinomas (due to the inactivation of both alleles of the *APC* gene) [59] and to poorly differentiated TCs and ATCs [56]. Since *APC* and *AXIN1* mutations are found in ATCs, but not in common PTCs, the molecular mechanisms that lead to the cytoplasmic stabilisation of β-catenin in the latter have not been yet established [58]. We observed the membranous expression of β-catenin in all normal, hyperplastic and tumour tissues, except for MSO and surrounding benign teratomas, which also presented cytoplasmic expression. Notably, the LOH for *AXIN1* was detected in both benign and malignant (MSO) teratoma tissues from this patient, contrarily to its eutopic thyroid tissues. In ATC, it has been hypothesised that LOH of the *AXIN1* gene can be associated with β-catenin accumulation [32]. However, this complete *AXIN1* inactivation in the benign and malignant teratoma tissues, in combination with *FOXE1* c.-522G>C, seemingly necessary, was not enough to induce malignant teratoma development, because a pathogenic somatic *BRAF* variant (p.Gly469Ala) was exclusively present in the MSO sample. As in eutopic thyroid gland carcinomas, the most common genetic alterations in MSO are *BRAF* abnormalities. Remarkably, the *BRAF* p.Gly469Ala variant was detected in 4/18 MSO cases in one study [37] and in two additional MSO cases [35,36], being considered the most common *BRAF* mutation in this malignancy [37]. This specific *BRAF* mutation is rarely found in eutopic thyroid gland carcinomas [60]. Interestingly, this patient’s MSO also presented a somatic gain at chromosome 3, where the *RAF1*, *CTNNB1*, *PIK3CA* and *DCUN1D1* genes are located. 

Overall, the results of this study reinforce the role of *FOXE1* variants in cases of thyroid ectopy, cleft palate and thyroid cancer. *AXIN1*, a major player in the Wnt/β-catenin signalling pathway, might also have played a role in the ectopic thyroid location. The genomic instability driven by these germline variants possibly contributed to the occurrence of somatic alterations, and to MSO and TC development, through MAPK pathway and PI3K signalling activation. 

A limitation of this work was the low number of cases analysed. Therefore, the study of additional cases of malignant struma ovarii, thyroid cancer and cleft palate will help to better understand the relevance of *AXIN1* and *FOXE1* in thyroid ectopy and malignancy. 

## 4. Materials and Methods

### 4.1. Patients

Two families followed in the Endocrinology Department of IPOLFG were analysed in the present study (Figure 1). 

In family 1 (F1), the proband (III.1) was diagnosed at the age of 20 with malignant struma ovarii (MSO), with a papillary cancer morphology with solid, trabecular and follicular patterns, representing about 50% of the partially cystic tumour. The tumour was excised laparoscopically in another institution and removed in multiple fragments, the largest measuring 60 mm. The patient underwent total thyroidectomy in the same year, which revealed thyroid follicular nodular disease (FND) and thyroiditis, and radioactive iodine. At the last follow-up, 10 years after the diagnosis, the patient had stable biochemical evidence of disease. Her mother (II.2) was asymptomatic, and the maternal grandmother (I.2), already deceased, had FND. 

In family 2 (F2), the proband (III.2) was diagnosed with an encapsulated follicular variant of PTC (fvPTC) and a septate uterus, having undergone a right hemithyroidectomy at the age of 19. Her sister (III.1), mother (II.2) and maternal uncle (II.3) had cleft palate (CP), and her maternal grandmother (I.2) presented hypothyroidism. 

DNA from the peripheral blood leukocytes of five affected and unaffected/asymptomatic family members, and formalin-fixed paraffin-embedded (FFPE) thyroid tissue samples of the F1 and F2 probands, and three unrelated thyroid cancer cases from our cohort, were available for study. A total of 100 DNA samples of peripheral blood leukocytes from healthy controls were also used (60% females and 40% males; median age 64 years, samples supplied by Biobanco-iMM, Lisbon Academic Medical Center, Lisbon, Portugal).

### 4.2. Nucleic Acid Extraction

Leukocyte DNA was extracted and purified using the Puregene^®^ Blood Core Kit (Qiagen, Hilden, Germany), according to the manufacturer’s protocol. DNA and RNA from FFPE tissues were extracted using the Maxwell^®^ RSC DNA FFPE Kit and Maxwell^®^ RSC RNA FFPE Kit (Promega, Madison, WI, USA), respectively, according to the manufacturer’s protocol, with minor alterations. Extracted nucleic acids were quantified using a Qubit 2.0 Fluorometer (Thermo Fisher Scientific, Wilmington, DE, USA).

### 4.3. Sanger Sequencing Analysis

The single *FOXE1* coding exon was amplified by PCR, and DNA sequencing was performed (ABI 3500; Applied Biosystems, Foster City, CA, USA). Additionally, variants located centromeric to *FOXE1* (rs965513 and rs7850258), at the *FOXE1* promoter (rs7849497, rs890127391, rs1867277, rs1867278, rs1867279, rs1867280 and rs911627696) and in the 3′UTR region (rs7046645) were studied.

In the *TERT* promoter (*TERTp*) region, where the most common mutations (c.−124C>T and c.-146C>T) are located, the read coverage obtained by next-generation sequencing (NGS) was very low in all samples. Thus, this analysis was performed by Sanger sequencing. 

Sanger sequencing was also used to validate some NGS findings and perform segregation studies in the families. Primer sequences and PCR conditions are available on request.

### 4.4. Next-Generation Sequencing (NGS)

The nucleic acids extracted from the blood and FFPE tissues of both families’ probands were selected for NGS analyses. The commercial AmpliSeq for Illumina Focus Panel (Illumina, San Diego, CA, USA) and a custom-made multigene panel [61] (SureSelect^XT HS^ Target Enrichment System for Illumina Multiplexed Sequencing Platforms; Agilent Technologies, Santa Clara, CA, USA) were used to detect somatic and germline alterations, respectively. Libraries were subjected to cluster generation on flow cell and paired-end sequencing in a MiSeq sequencer platform (Illumina).

For the identification of somatic variants, bioinformatics analysis was carried out using the software DNA + RNA Amplicon v.1.0.5 (Illumina), for the alignment and analysis of fusions in target genes, and Variant Interpret v.2.16.2.1 (Illumina), for the analysis and annotation of genetic variants. For the study of germline alterations, the Agilent SureCall version 4.2.2.3 and Alissa Interpret software version 5.4.2 programs (Agilent Technologies) were used. Multiple in silico prediction tools were used for variant classification (https://franklin.genoox.com—Franklin by Genoox): REVEL, AlphaMissense, EVE, MutationAssessor, SIFT, PolyPhen-2, MutationTaster, DANN, MetaLR, PrimateAI, BayesDel, GERP, GenoCanyon, fitCons.

### 4.5. Quantitative Reverse Transcription PCR (RT-qPCR)

cDNA was synthesised from 1 μg of total RNA, and RT-qPCR was conducted in an Applied Biosystems QuantStudio^TM^ 5 Real-Time PCR System (Applied Biosystems, Waltham, MA, USA). Reactions were performed in triplicate using the TaqMan Universal PCR Master Mix (Applied Biosystems, Waltham, MA, USA) for 50 cycles. Relative gene expression levels were quantified using the comparative threshold cycle 2^−ΔΔCt^ method (Livak and Schmittgen 2001) by normalising target gene (*FOXE1*) levels to the expression of a housekeeping gene (*YWHAZ*), employing TaqMan probes (TaqMan^®^ Gene Expression Assay ID Hs00916085_s1 for FOXE1 and Hs0112247_g1 for YWHAZ) (Applied Biosystems). 

### 4.6. Immunohistochemistry (IHC)

The immunohistochemical detection of FOXE1 and β-catenin proteins was performed on archival FFPE tumour samples. A total of 5 thyroid cancer cases, previously assessed for *FOXE1* germline mutational status, were included: probands from F1 (III.1) and F2 (III.2), and three *FOXE1* wild-type (WT) patients. The adjacent normal tissue in each tumour sample, as well as hyperplastic tissue in those patients presenting thyroid FND, were also analysed. 

IHC was performed on an automated Ventana^®^ Benchmark ULTRA system (Roche Diagnostics, Basel, Switzerland) with the Ventana^®^ Optiview DAB IHC detection kit (ref. 760–700; Roche Diagnostics, Basel, Switzerland). The FOXE1 polyclonal antibody (orb522397; Biorbyt Ltd., Cambridge, UK) at 1:100 dilution was incubated for 32 min; antigen retrieval was accomplished with a low pH solution. The β-catenin mouse monoclonal antibody (clone 14, ref 760–4242; Roche Diagnostics) was incubated for 24 min; antigen retrieval was performed with CC1 (40 min at 98 °C). IHC results were interpreted in terms of expression (positive or negative), staining intensity (strong, moderate or weak), extent (focal or diffuse) and/or subcellular localisation (nuclear, cytoplasmic and membranous). 

### 4.7. Cell Culture

Two distinct cell lines were used in this study: a human PTC cell line (TPC-1; a kind gift from Dr. Paula Soares, IPATIMUP, Porto, Portugal) and *Rattus norvegicus* normal thyroid cells (PCCL3; kindly provided by Prof. Jacques Dumont, Université Libre de Bruxelles, Belgium). TPC-1 and PCCL3 cells were cultured as previously described [55]. 

### 4.8. Constructs, Transient Transfections and Luciferase Reporter Gene Assays

For the functional studies, a 1.30-kb fragment including the 5′ upstream regulatory region of the human *FOXE1* gene [−985, +256 bp; relative to the transcriptional start site (+1)] was incorporated into a pGL3-Basic plasmid, upstream of luciferase. *FOXE1* variants (c.-522G>C and c.9C>T) were generated by site-directed mutagenesis. The constructs were validated by Sanger sequencing.

For the luciferase reporter assays, PCCL3 and TPC-1 cells were plated in a 24-well culture plate at a density of 5 × 10^4^ and 7.5 × 10^4^ cells/well, respectively, 24 h before transfection. Then, cells were transiently transfected using Lipofectamine 2000 Transfection Reagent (Thermo Fisher Scientific, Waltham, MA, USA) together with 500 ng Firefly Luciferase Reporter Vector or an empty expression vector control and 10 ng of SV40 Renilla Luciferase Reporter Vector, to assess the transfection efficiency. After 48 h, cells were harvested, lysed and analysed for luciferase and Renilla activity, using the Dual-Luciferase Reporter Assay System (PJK GmbH, Kleinblittersdorf, Germany). At least three independent experiments were performed.

### 4.9. Statistical Analysis

All data are reported as mean ± standard deviation. Comparisons between two groups were made using a two-tailed Student’s unpaired *t*-test. Statistical analysis was performed with the GraphPad Prism version 7.0 (GraphPad Software, Boston, MA, USA). Differences were considered statistically significant at *p* ≤ 0.05.

## Figures and Tables

**Table 1:** Summary of the genetic alterations identified in the leukocytes and tumours from the probands of families F1 and F2.

	Gene	Status	DNA	Protein	dbSNP ID	MAF (%) ^a^	Variant’s Predicted Impact ^b^
**F1: III.1**	*FOXE1*	Germline	c.-522G>C	-	rs890127391	0.006	VUS
	*AXIN1*	Germline	c.1121C>G	p.Thr374Arg	n/a	n/a	VUS
	*TERTp*	Germline	c.-245T>C	-	rs2853669	30.6	Polymorphism
	*BRAF*	Somatic	c.1406G>C	p.Gly469Ala	rs121913355	0.0	Deleterious (10/14) ^c^
	*RAF1*	Somatic	Gain				
	*CTNNB1*	Somatic	Gain				
	*PIK3CA*	Somatic	Gain				
	*DCUN1D1*	Somatic	Gain				
**F2: III.2**	*FOXE1*	Germline	c.9C>T	p.Ala3=	rs911627696	0.006	VUS
	*HRAS*	Somatic	c.182A>G	p.Gln61Arg	rs121913233	0.0	Deleterious (10/14) ^c^

^a^ Information for MAF (minor allele frequency) refers to non-Finnish European (NFE) population from the Genome Aggregation Database (gnomAD v4.0.0). ^b^ Detailed in Tables S2 and S4. ^c^ In silico prediction tools: REVEL, AlphaMissense, EVE, MutationAssessor, SIFT, PolyPhen-2, MutationTaster, DANN, MetaLR, PrimateAI, BayesDel, GERP, GenoCanyon, fitCons. VUS, variant of uncertain significance; n/a, not available.

## Data Availability

The data generated during and/or analysed during the current study are available from the corresponding author on reasonable request.

## Supplementary Materials

The following supporting information can be downloaded at: https://www.mdpi.com/article/10.3390/ijms25041966/s1.
