# Supplementary material for: Identification of Germline FOXE1 and Somatic MAPK Pathway Gene Alterations in Patients with Malignant Struma Ovarii, Cleft Palate and Thyroid Cancer
Source: Int J Mol Sci. 2024 Feb 6;25(4):1966. doi: 10.3390/ijms25041966 (PMC10888156; doi:10.3390/ijms25041966)
Supplement: Supplementary file 1 [file ijms-25-01966-s001.zip › ijms-2809700-supplementary.pdf]

Identification of Germline *FOXE1* and Somatic *MAPK* Pathway Gene Alterations in Patients with Malignant Struma Ovarii, Cleft Palate and Thyroid Cancer

Carolina Pires, Ana Saramago, Margarida M. Moura, Jing Li, Sara Donato, Inês J. Marques, Hélio Belo, Ana C. Machado, Rafael Cabrera, Thomas G. P. Grünewald, Valeriano Leite, Branca M. Cavaco

Supplementary Materials

Tables

Table S1. FOXE1 expression in thyroid tumours and adjacent-normal and hyperplastic patients' tissues.

		FOXE1 immunohistochemical staining pattern		
	**Tissue type**	**Expression**	**Staining intensity**	**Extent**
**Patient 1** *FOXE1* WT	T	Negative	-	-
	N	Positive	Moderate	Focal
	H	Positive	Moderate	Focal
**Patient 2** *FOXE1* WT	T	Negative	-	-
	N	Positive	Weak	Focal
**Patient 3** *FOXE1* WT	T	Negative	-	-
	N	Positive	Weak	Focal
	H	Positive	Weak	Focal
**F1: Patient III.1** *FOXE1* c.-522G>C	T[a]	Negative	-	-
	N[a]	Positive	Moderate	Focal
	N[b]	Positive	Weak	Focal
	H[b]	Positive	Weak	Focal
**F2: Patient III.2** *FOXE1* c.9C>T	T	Negative	-	-
	N	Positive	Moderate	Focal

T, tumour tissue; N, normal tissue; H, hyperplastic tissue; WT, wild-type. [a] refers to benign and malignant thyroid tissues derived from the ovary teratoma, while [b] refers to the eutopic thyroid tissues from the same patient.

Table S2. List of germline variants identified in F1 and F2 probands.

	Gene	DNA	Protein	dbSNP ID	MAF (%)	ClinVar interpretation	ACMG
**F1: III.1**	*FOXE1*	c.-522G>C	-	rs890127391	0.006	n/a	VUS[a]
	AXIN1	c.1121C>G	p.Thr374Arg	n/a	n/a	n/a	VUS[b]
	TERTp	c.-245T>C	-	rs2853669	30.6	Benign	Benign
**F2: III.2**	*FOXE1*	c.9C>T	p.Ala3=	rs911627696	0.006	n/a	VUS[a]

[a] PM2; [b] PM2, PP3. ACMG, American College of Medical Genetics and Genomics; VUS, variant of uncertain significance; n/a, not available.

Table S3. β-Catenin expression in thyroid tumours and adjacent normal and hyperplastic patients' tissues.

	**β-Catenin immunohistochemical staining pattern**			
		**Subcellular localization**		
	**Tissue type**	**Membranous**	**Cytoplasmic**	**Nuclear**
**Patient 1**	T	Positive	Negative	Negative
	N	Positive	Negative	Negative
	H	Positive	Negative	Negative
**Patient 2**	T	Positive	Negative	Negative
	N	Positive	Negative	Negative
**Patient 3**	T	Positive	Negative	Negative
	N	Positive	Negative	Negative
	H	Positive	Negative	Negative
**F1: Patient III.1***	T[a]	Positive	**Positive**	Negative
	N[a]	Positive	**Positive**	Negative
	N[b]	Positive	Negative	Negative
	H[b]	Positive	Negative	Negative
**F2: Patient III.2**	T	Positive	Negative	Negative
	N	Positive	Negative	Negative

T, tumour tissue; N, normal tissue; H, hyperplastic tissue. [a] refers to benign and malignant thyroid tissues derived from the ovary teratoma, while [b] refers to the eutopic thyroid tissues from the same patient. *Carrier of germline *AXIN1* p.Thr374Arg variant.

Table S4. List of somatic variants identified in F1 and F2 probands.

	Gene	DNA	Protein	dbSNP ID	MAF (%)	COSMIC	AMP Classification	*In silico* prediction
**F1: III.1**	*BRAF*	c.1406G>C	p.Gly469Ala	rs121913355	0.0	COSV56061424	Tier 1	Deleterious (10/14)[a]
**F2: III.2**	*HRAS*	c.182A>G	p.Gln61Arg	rs121913233	0.0	COSV54236691	Tier 1	Deleterious (10/14)[b]

[a] [https://franklin.genoox.com/clinical-db/variant/snpTumor/chr7-140481402-C-G?app=assessment-tools (assessed on 5 February 2024)];

[b] [https://franklin.genoox.com/clinical-db/variant/snp/chr11-533874-T-C-hg38?app=assessment-tools (accessed on 5 February 2024)]. AMP, Association for Molecular Pathology.

Figures

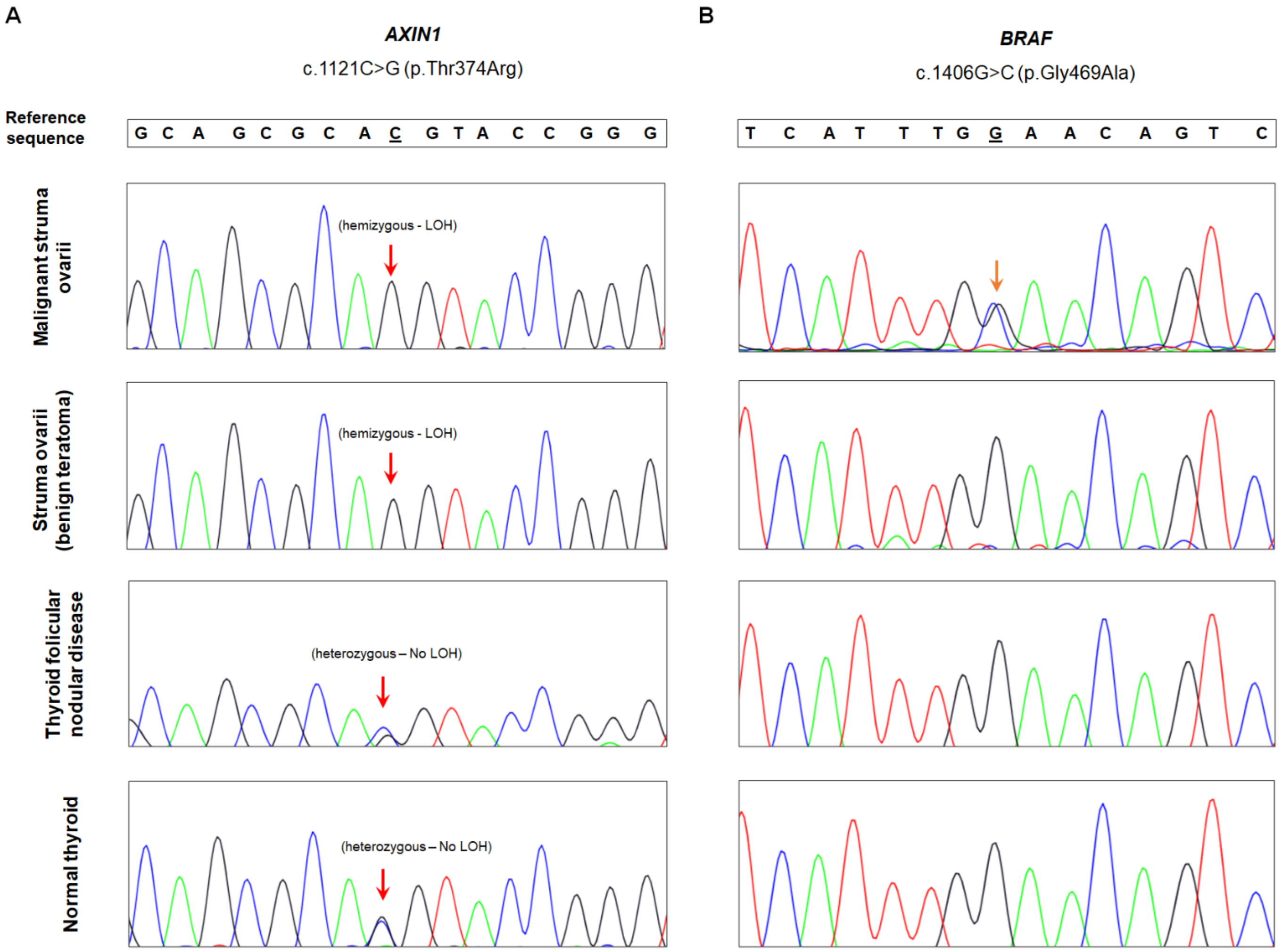

Figure S1. Sanger sequencing analyses of *AXIN1* (A) and *BRAF* (B) variants in the tissues from the proband (III.1) of Family 1 (F1). A germline *AXIN1* variant (p.Thr374Arg) was detected in heterozygosity in this patient and asymptomatic mother. In benign thyroid lesions (follicular nodular disease) and normal thyroid tissues both wild-type (allele C) and mutant (allele G; red arrow) alleles are present, but in benign and malignant teratoma tissues there is a loss of the wild-type allele [loss of heterozygosity (LOH)]. A somatic *BRAF* pathogenic variant (p.Gly469Ala) was only detected in the malignant struma ovarii sample (orange arrow).
